# Erythropoietin Protects Against Lipopolysaccharide-Induced Microgliosis and Abnormal Granule Cell Development in the Ovine Fetal Cerebellum

**DOI:** 10.3389/fncel.2017.00224

**Published:** 2017-07-28

**Authors:** Annie R. A. McDougall, Nadia Hale, Sandra Rees, Richard Harding, Robert De Matteo, Stuart B. Hooper, Mary Tolcos

**Affiliations:** ^1^The Ritchie Centre, Hudson Institute of Medical Research Clayton, VIC, Australia; ^2^Department of Obstetrics and Gynaecology, Monash University Clayton, VIC, Australia; ^3^Department of Anatomy and Neuroscience, University of Melbourne Parkville, VIC, Australia; ^4^Department of Anatomy and Developmental Biology, Monash University Clayton, VIC, Australia; ^5^School of Health and Biomedical Sciences, RMIT University Melbourne, VIC, Australia

**Keywords:** chorioamnionitis, inflammation, brain development, cell proliferation, neuronal migration

## Abstract

Erythropoietin (EPO) ameliorates inflammation-induced injury in cerebral white matter (WM). However, effects of inflammation on the cerebellum and neuroprotective effects of EPO are unknown. Our aims were to determine: (i) whether lipopolysaccharide (LPS)-induced intrauterine inflammation causes injury to, and/or impairs development of the cerebellum; and (ii) whether recombinant human EPO (rhEPO) mitigates these changes. At 107 ± 1 days gestational age (DGA; ~0.7 of term), fetal sheep received LPS (~0.9 μg/kg; i.v.) or an equivalent volume of saline, followed 1 h later with 5000 IU/kg rhEPO (i.v.) or an equivalent volume of saline (i.v.). This generated the following experimental groups: control (saline + saline; *n* = 6), LPS (LPS + saline, *n* = 8) and LPS + rhEPO (*n* = 8). At necropsy (116 ± 1 DGA; ~0.8 of term) the brain was perfusion-fixed and stained histologically (H&E) and immunostained to identify granule cells (Neuronal Nuclei, NeuN), granule cell proliferation (Ki67), Bergmann glia (glial fibrillary acidic protein, GFAP), astrogliosis (GFAP) and microgliosis (Iba-1). In comparison to controls, LPS fetuses had an increased density of Iba-1-positive microglia (*p* < 0.005) in the lobular WM; rhEPO prevented this increase (*p* < 0.05). The thickness of both the proliferative (Ki67-positive) and post-mitotic zones (Ki67-negative) of the EGL were increased in LPS-exposed fetuses compared to controls (*p* < 0.05), but were not different between controls and LPS + rhEPO fetuses. LPS also increased (*p* < 0.001) the density of granule cells (NeuN-positive) in the internal granule layer (IGL); rhEPO prevented the increase (*p* < 0.01). There was no difference between groups in the areas of the vermis (total cross-section), molecular layer (ML), IGL or WM, the density of NeuN-positive granule cells in the ML, the linear density of Bergmann glial fibers, the areal density or somal area of the Purkinje cells, the areal coverage of GFAP-positive astrocytes in the lobular and deep WM, the density of Iba-1-positive microglia in the deep WM or the density of apopotic cells in the cerebellum. LPS-induced intrauterine inflammation caused microgliosis and abnormal development of granule cells. rhEPO ameliorated these changes, suggesting that it is neuroprotective against LPS-induced inflammatory effects in the cerebellum.

## Introduction

Preterm babies have an increased risk of neurodevelopmental delay, disability, sensory and learning deficits and cerebral palsy (Beck et al., 2010; Doyle et al., 2010), and deficits are more pronounced the earlier that infants are born (Saigal and Doyle, 2008). Advances in perinatal care have resulted in an increasing number of very preterm babies surviving and hence the number of infants with potential perinatal brain injury and disability is increasing. Protecting the brain of preterm infants from injury is one of the greatest challenges of perinatal medicine. The two major causes of brain injury prior to, or around the time of birth, are inflammation (such as inflammation of the fetal membranes, termed chorioamnionitis; Dammann and Leviton, 1997, 2004) and/or cerebral hypoxia (Volpe, 2001). There is a strong causal relationship between chorioamnionitis and preterm birth (Lahra and Jeffery, 2004), and preterm birth increases the risk and severity of neonatal brain injury (Yoon et al., 1996, 1997). Infants who have been exposed to maternal or intrauterine infection and inflammation have an increased risk of diffuse cerebral white matter (WM) injury, intraventricular hemeorrhage, periventricular leukomalacia and cerebral palsy (Grether and Nelson, 1997; Goldenberg et al., 2000; Wu and Colford, 2000; Leviton et al., 2005; Romero et al., 2006). Thus, although the effects of inflammation on the human cerebrum are well established, far less attention has been paid to any injury and adverse development sustained by the cerebellum.

The cerebellum develops rapidly in the second half of gestation and during early post-natal life in all long gestation species including humans (Friede, 1973), making it potentially vulnerable to perinatal insults, such as inflammation. Although it is known that there are adverse effects on cerebellar development in infants who have also sustained supratentorial damage (Shah et al., 2006; Srinivasan et al., 2006), few studies have specifically examined the effects of inflammation on the developing human cerebellum. There is evidence, however, from animal studies, largely in sheep, that the fetal cerebellum is vulnerable to inflammation induced by lipopolysaccharide (LPS). Inflammatory damage has been reported to include: cerebellar WM injury and focal WM lesions (Dean et al., 2009), increased expression of pro-inflammatory cytokines (Strackx et al., 2015), apoptosis (Hutton et al., 2007; Gavilanes et al., 2009), increased activation of microglia (Hutton et al., 2007; Gavilanes et al., 2009), infiltration of macrophages (Hutton et al., 2007), astrogliosis (Hutton et al., 2007; Gavilanes et al., 2009; Strackx et al., 2012), an increase in the number of granule neurons in the internal granule layer (IGL; Strackx et al., 2012), a reduction in the proportion of mature oligodendrocytes (Dean et al., 2009; Gavilanes et al., 2009) and breakdown of the blood-brain barrier (Hutton et al., 2007). In a previous study on the effects of LPS on the fetal sheep brain (Duncan et al., 2002), we found significant injury in the cerebrum but no gross cerebellar damage; as we recognize that damage could have occurred at the cellular level, we have addressed this possibility in the present study.

The cytokine hormone erythropoietin (EPO) has extensive neuroprotective effects, via its anti-inflammatory, anti-apoptotic and anti-oxidative stress actions (van der Kooij et al., 2008). We have previously shown that recombinant human (rh)EPO is protective against LPS-induced brain injury in the fetal sheep cerebrum, reducing cerebral WM injury, apoptosis, microgliosis, astrogliosis and blood-brain barrier leakage (Rees et al., 2010). rhEPO is currently being tested in clinical trials for its neuroprotective effects in preterm infants, with initial results suggesting that rhEPO improves neurological outcomes (Neubauer et al., 2010) and reduces cerebral WM damage (O’Gorman et al., 2015). The neuroprotective effects of rhEPO on the cerebellum remain unknown. In the present study, we tested the hypothesis that rhEPO would prevent or ameliorate injury or altered development in the cerebellum of our well established model of endotoxin- induced inflammation of the ovine fetal brain (Duncan et al., 2002; Rees et al., 2010).

## Materials and Methods

All animal procedures were approved by the Monash University Animal ethics committee.

### Surgery

Time-mated pregnant Merino × Border Leicester ewes (*n* = 19) underwent sterile surgery at 102 ± 1 day of gestational age (DGA; term ~147 days) to chronically implant catheters into a fetal femoral artery (for fetal monitoring) and vein (for drug delivery). Ampicillin sodium (1 g/5 ml H_2_O, intramuscular, Optigen Ingredients, Port Adelaide, SA, Australia) was administered to all ewes for 3 days after surgery. Fetal blood was sampled on the day after surgery and then every 2 days until the end of the experiment. Fetal blood-gases and proinflammatory cytokine levels have been previously published (Rees et al., 2010).

### Experimental Protocol

At 107 ± 1 DGA, fetuses were randomly assigned to receive intravenous LPS (~0.9 μg/kg estimated fetal weight; Escherichia coli, 0.55:B55; Sigma Chemical, St Louis, MO, USA) or an equivalent volume of saline. One hour later, we administered intravenous rhEPO (5000 IU/kg estimated fetal body weight, Epoetin-α; Janssen-Cilag, Macquarie Park, NSW, Australia) or an equivalent volume of saline. Study groups were as follows: saline followed by saline (control, *n* = 6), LPS followed by saline (LPS, *n* = 8) and LPS followed by rhEPO (LPS + rhEPO, *n* = 8). The treatments were repeated for three consecutive days. Fetal physiological measurements were recorded throughout the experimental protocol and have been published; we previously found a significant fetal hypoxemia following LPS administration, in both LPS and LPS + rhEPO fetuses (Rees et al., 2010).

### Tissue Preparation

The ewe and fetus were killed with an overdose of sodium pentobarbitone (130 mg/kg, intravenous) administered to the ewe at 116 ± 1 DGA. The fetus was weighed and the brain immediately perfused with 4% paraformaldehyde (PFA) in 0.1 M phosphate-buffered saline (PBS; pH 7.4). The brain was dissected from the skull and post-fixed in 4% PFA/0.1 M PBS for 4 h at 4°C. The cerebellum was removed from the brain at the level of the cerebellar peduncles and bisected at the mid-line of the vermis. The left side of the cerebellum was processed to paraffin wax and sagittally sectioned at 8 μm for immunohistochemistry and H&E staining.

### Immunohistochemistry

Immunohistochemistry was performed on paraffin-embedded sections at the level of the cerebellar vermis to detect neuron-specific protein NeuN (NEUronal nuclei; granule cells, mouse α-NeuN, 1:500; Millipore, USA; Weyer and Schilling, 2003), Ki67 (cells proliferating in late G1, S, G2 and M phases of cell cycle, mouse α-Ki67, 1:500, Thermo Scientific, USA), glial fibrillary acidic protein (GFAP; Bergmann glial fibers and astrocytes, rabbit α-GFAP, 1:1000, DAKO, USA) and ionized calcium binding adapter molecule-1 (Iba-1; microglia, rabbit α-Iba-1, 1:1500, WAKO Pure Chemical Industries, Japan) using biotinylated mouse or rabbit α-IgG secondary antibodies (1:200) and the avidin-biotin complex elite kit (Vector Laboratories, Burlingame, CA, USA) as previously described (Rees et al., 2010; Tolcos et al., 2011). When the primary antibody was omitted, immunoreactivity failed to occur. For each antibody, sections from the three treatment groups were stained simultaneously to ensure uniform conditions for subsequent analysis.

### Quantitative Analysis

Analyses were performed on coded slides (observer blinded to group) from the cerebellum at the level of the vermis using image analysis software (ImageScope, Aperio technologies, Vista, CA, USA or ImageJ software)^1^. All analysis was performed in randomly selected fields in two sections/animal, spaced 80 μm apart. Means were calculated for each animal and a mean of means for each treatment group was determined; all densities are expressed as cells/mm^2^.

#### Analysis of H&E Sections

H&E-stained sections at the level of the vermis were scanned using an Aperio slide scanner (Leica Biosystems, Germany) and analyzed using ImageScope software. The total cross-sectional areas of the vermis and deep WM were measured from tracing outlines of these regions. The areas of the molecular layer (ML), IGL and lobular WM were measured from tracings of these regions in early (lobule X) and late (lobule VIII) developing lobules (Altman, 1969). Due to the difficulty in preserving the entire external granule layer (EGL) within a lobule, the width, rather than the area of the EGL in both lobules VIII and X was calculated in sections stained with Ki67 (see below).

The linear density of H&E-stained Purkinje cells was estimated by counting all cell profiles that intersected a horizontal line of known length and then dividing the number of cells by the length of the line. Counts were made in four random fields of view in both lobules X and VIII, in two sections from each animal (total: 16 fields of view per animal). The areal density of Purkinje cells was then calculated by dividing the number of Purkinje cells/mm of Purkinje cell line by the section thickness (=mean diameter of the Purkinje cell plus section thickness; Rees et al., 1997). In all groups, mean diameter of the cell soma was 22.4 μm. The somal area was measured in 20 cells in each lobule (X and VIII), in two sections per animal (40 cells counted per lobule across the two sections). Only cells in which the nucleus and nucleolus were visible were measured for somal area and the calculation of the somal diameter.

#### Analysis of Immunostained Sections

The widths of the Ki67-positive (proliferative) and the Ki67-negative (post-mitotic) zones of the EGL were calculated by measuring the length and area of eight segments of each layer per section (four segments in lobule X and four segments in lobule VIII) and dividing the area by the length of each segment. For each animal, mean widths were determined for each lobule; these values were combined and a group mean calculated.

The areal density of NeuN-positive granule cells was determined in the ML (three fields of view per lobule X and VIII; total area counted was 40 μm^2^ with a minimum of 600 cells counted per animal) and IGL (two fields of view per lobule X and VIII; total area counted was 40 μm^2^ per animal with a minimum 800 cells counted per animal). The areal density of NeuN-positive granule cells (cells/mm^2^) in the ML and IGL was then determined by dividing the number of cells by the area of the field of view.

The linear density of the Bergmann glial fibers (GFAP-positive fibers) was determined using ImageJ. A horizontal line (0.1 mm in length) was drawn across the image of the cerebellum, perpendicular to, and approximately midway along, the length of the Bergmann glial fibers. Fibers that intersected the line were counted in two random fields of view in both lobules X and VIII, in two sections from each animal (total: eight fields of view per animal). The linear density of fibers (cells/mm) was then determined by dividing the number of fibers by length of the line.

Areal coverage of GFAP-positive astrocytes and their processes in the cerebellar WM was quantified. For each section, three fields of view (0.04 mm^2^) were taken from lobule X and lobule VIII, as well as the deep WM (nine fields in total). The proportion (%) of cerebellar WM (lobular or deep WM) occupied by GFAP-positive astrocytes (i.e., areal coverage) was determined using ImageJ Fiji Analysis software.

To determine the density of microglia in the cerebellar WM, sections were digitally scanned (Image Scope, Aperio Technologies Inc., Germany) and analyzed at 400× magnification. Iba-1-positive cells were manually counted in three fields (0.04 mm^2^) taken from lobules X and VIII, as well as the deep WM (nine fields in total). The density (cells/mm^2^) was then determined by dividing the number of cells by the area of the field of view.

#### TUNEL Staining and Analysis

Apoptotic cells were identified using the DeadEnd™ Colormetric TUNEL system on paraffin-embedded tissue sections, according to manufacturer’s instructions (G7130, Promega, Australia). The density of apoptotic cells was determined by counting the number of TUNEL-positive cells in the total cross-sectional areas of the ML, IGL, deep WM and lobular WM (X and VIII). The area of each of the regions was measured and data expressed as TUNEL-positive cells/mm^2^. Cell densities in lobule X and VIII were combined and then averaged across two sections per animal. In the EGL, the density of TUNEL-positive cells could not be calculated due to difficulties encountered in measuring the entire EGL area in immunoreacted tissue. However, small numbers of TUNEL-positive cells were observed in the EGL in all groups and qualitatively no overt differences were observed between groups.

### Statistical Analysis

All data were reported as mean ± standard error of the mean (SEM), except for brain and cerebellar weight, which are expressed as mean ± standard deviation. All analyses were performed using a one-way ANOVA in GraphPad Prism 6 (GraphPad Software, Inc., CA, USA). When statistical differences were found, a Bonferroni *post hoc* test was performed to identify differences between each group. Data was considered significant when *p* < 0.05.

## Results

### Brain Weights

There was no difference (*p* > 0.05) in brain weight, cerebellar weight or cerebellar to body weight ratio between control, LPS or LPS + rhEPO animals (Table 1).

**Table 1:** Body and brain weights (mean ± SD).

	Control *n* = 6	LPS *n* = 8	LPS + rhEPO *n* = 8
Body weight (kg)	2.0 ± 0.1	2.0 ± 0.1	2.1 ± 0.1
Brain weight (g)	32.8 ± 0.1	33.9 ± 1.1	32.2 ± 1.8
Cerebellar weight (g)	2.5 ± 0.1	2.5 ± 0.1	2.4 ± 0.2
Cerebellar: body weight	1.3 ± 0.1	1.3 ± 0.1	1.1 ± 0.1

### Morphology of the Cerebellum

**Figure 1:**
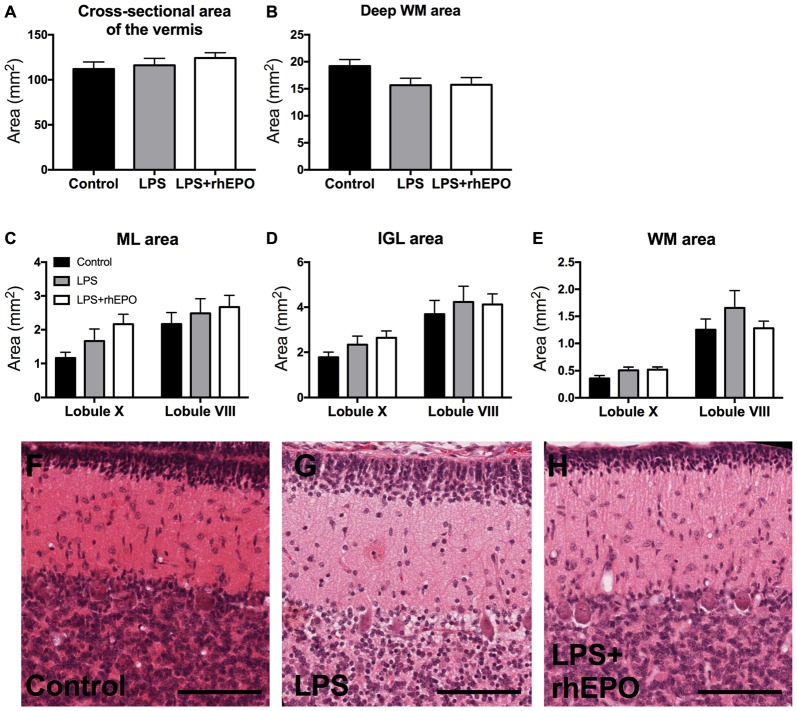
Morphology of the cerebellum. **(A)** The total cross-sectional area of the cerebellar vermis, and the cross-sectional area of the **(B)** deep white matter (dWM), **(C)** molecular layer (ML), **(D)** internal granule layer (IGL) and **(E)** lobular WM in saline-treated Control (black bars), lipopolysaccharide (LPS)-treated (gray bars) and LPS + rhEPO-treated (white bars) fetal sheep. Data from the ML, IGL and lobular WM are presented from lobule X (early developing) and VIII (late developing). Representative images of H&E staining in **(F)** saline-treated (Control), **(G)** LPS-treated and **(H)** LPS + rhEPO-treated fetal sheep. Scale bar = 100 μm.

No hemeorrhages or infarcts were observed in the controls, LPS or LPS + rhEPO groups. There were no differences (*p* > 0.05) between controls, LPS or LPS + rhEPO groups in the total cross sectional area of the vermis (Figure 1A) or the deep WM (Figure 1B). Neither were there differences (*p* > 0.05) between groups in the areas of the ML, IGL and lobular WM in either lobule VIII or X (Figures 1C–H).

### Granule Cell Proliferation

**Figure 2:**
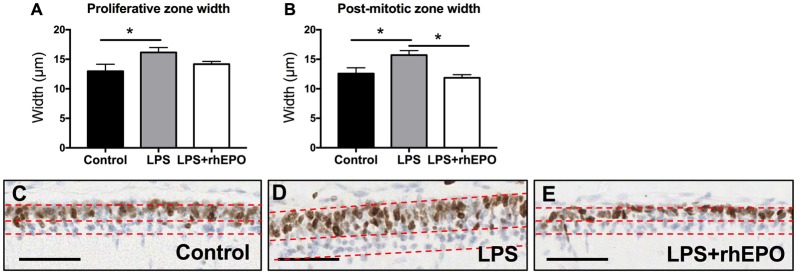
Proliferation in the external granule layer (EGL). Mean (±SEM) width of the **(A)** proliferative zone and **(B)** the post-mitotic zone in saline-treated (Controls; black bars), LPS-treated (gray bars) and LPS + rhEPO-treated (white bars) fetal sheep. *p < 0.05. Ki67 staining (brown nuclei) in the EGL of **(C)** saline-treated (Control), **(D)** LPS-treated and **(E)** LPS + rhEPO-treated fetal sheep. All images counterstained with hemeatoxylin (blue). Red dotted lines show the borders of the proliferative zone (brown stained cells, upper portion of the EGL) and the post-mitotic zone (blue stained cells, lower portion of the EGL). Scale bar = 50 μm.

The width of the proliferative zone (Ki67 positive) of the EGL in LPS animals was greater than in control animals (*p* = 0.048) but was not different (*p* > 0.05) to the LPS + rhEPO group (Figure 2A). The width of the post-mitotic zone (Ki67 negative) of the EGL was greater in LPS animals compared to controls and to LPS + rhEPO animals (*p* = 0.03; Figure 2B). This is illustrated in Figures 2C–E.

### Granule Cell Density

**Figure 3:**
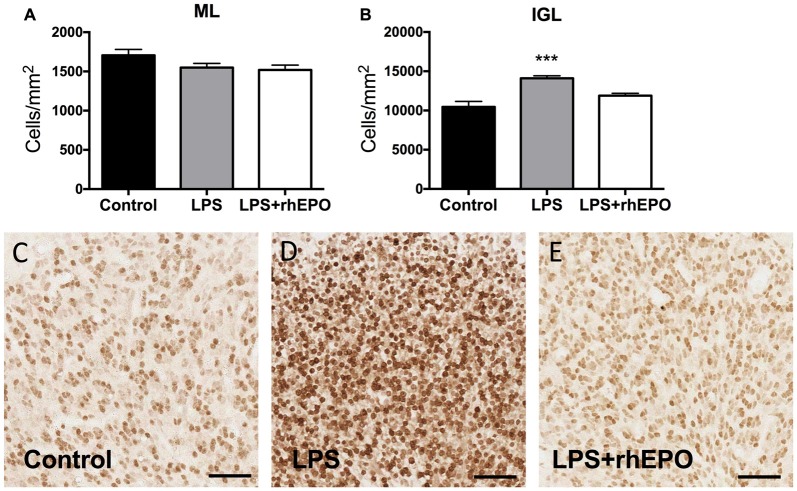
Granule cell density. The mean (±SEM) areal density of **(A)** NeuN-positive granule cells in the ML and **(B)** of NeuN-positive granule cells in the IGL of saline-treated (Controls; black bars), LPS-treated (gray bars) and LPS + rhEPO-treated (white bars) fetal sheep. ***p < 0.001, compared to Controls. NeuN-positive granule cells (brown) in the IGL in **(C)** saline-treated (Control), **(D)** LPS-treated and **(E)** LPS + rhEPO-treated fetal sheep. Scale bar = 50 μm.

The areal density of granule cells in the ML in LPS and LPS + rhEPO animals was not different (*p* > 0.05) from that of controls (Figure 3A). The density of granule cells in the IGL was greater in LPS (*p* = 0.0002) animals compared to controls, but was not different (*p* > 0.05) between control and LPS + rhEPO animals (Figures 3B,C–E).

### The Migratory Scaffold of the Cerebellum

**Figure 4:**
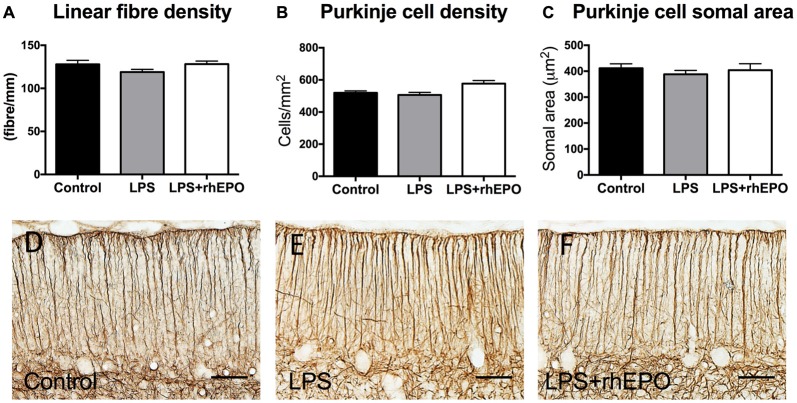
Bergmann glial fibers and Purkinje cells. **(A)** The mean (±SEM) linear density of glial fibrillary acidic protein (GFAP)-positive Bergmann glial fibers in the ML. The mean (±SEM) **(B)** areal density and **(C)** somal area of Purkinje cells in saline-treated Controls (black bars), LPS-treated (gray bars) and LPS + rhEPO-treated (white bars) fetal sheep. GFAP-positive Bergmann glial fibers (brown staining) in **(D)** saline-treated (Control), **(E)** LPS-treated and **(F)** LPS + rhEPO-treated fetal sheep. Scale bar = 50 μm.

There were no differences in the linear density of Bergmann glial fibers in the ML between control, LPS or LPS + rhEPO animals (*p* > 0.05; Figures 4A,D–F).

### Purkinje Cell Areal Density and Somal Area

There were no differences in the areal density of Purkinje cells between control, LPS or LPS + rhEPO groups (*p* > 0.05; Figure 4B). There was no difference in the Purkinje cell somal area between any of the groups (*p* > 0.05; Figure 4C).

### Microgliosis and Astrogliosis

**Figure 5:**
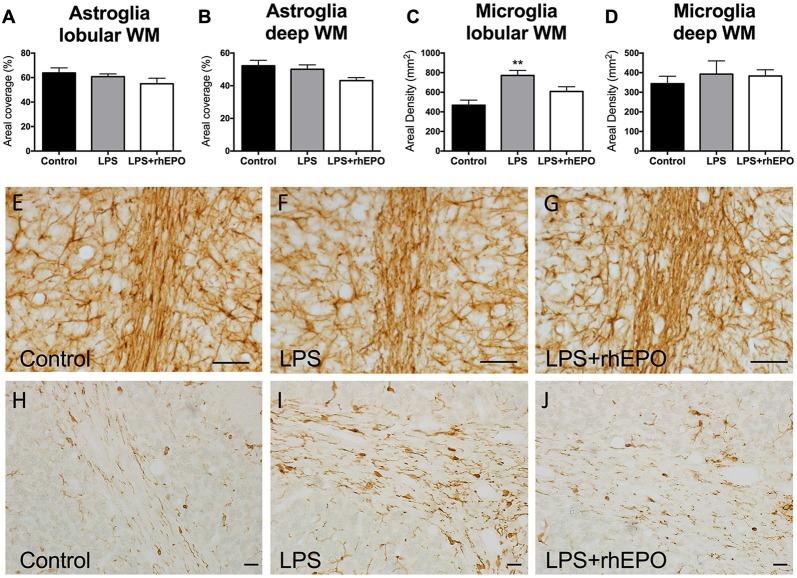
Astrogliosis and Microgliosis in the WM. The mean (±SEM) areal coverage (%) of GFAP-positive astrocyte staining in the **(A)** lobular WM and **(B)** deep WM (dWM) and the mean (±SEM) areal density of Iba-1-positive microglia staining in the **(C)** lobular WM and **(D)** dWM, in saline-treated Controls (black bars), LPS-treated (gray bars) and LPS + rhEPO-treated (white bars) fetal sheep. **p < 0.01 compared to Controls. GFAP-positive astrocytes (brown staining) in lobule VIII WM of **(E)** saline-treated (Control), **(F)** LPS-treated and **(G)** LPS + rhEPO-treated fetal sheep. Scale bar **(E–G)** = 10 μm. Iba-1-positive microglia in lobule VIII WM in **(H)** saline-treated (Control), **(I)** LPS-treated and **(J)** LPS + rhEPO-treated fetal sheep. Scale bar **(H–J)** = 20 μm.

There were no differences (*p* > 0.05) in the areal coverage of GFAP-positive astrocytes in the lobular WM (Figure 5A) or the deep WM (Figure 5B) between control, LPS or LPS + rhEPO animals (Figures 5E–G).

In the lobular WM, the density of Iba-1-positive microglia was greater in LPS animals compared to controls (*p* = 0.002), but was not different (*p* > 0.05) in LPS + rhEPO animals compared to controls (Figures 5C,H–J). There were no differences in the density of Iba-1-positive microglia in the deep WM between control, LPS or LPS + rhEPO animals (*p* > 0.05; Figure 5D).

### Apoptosis in the Cerebellum

**Figure 6:**
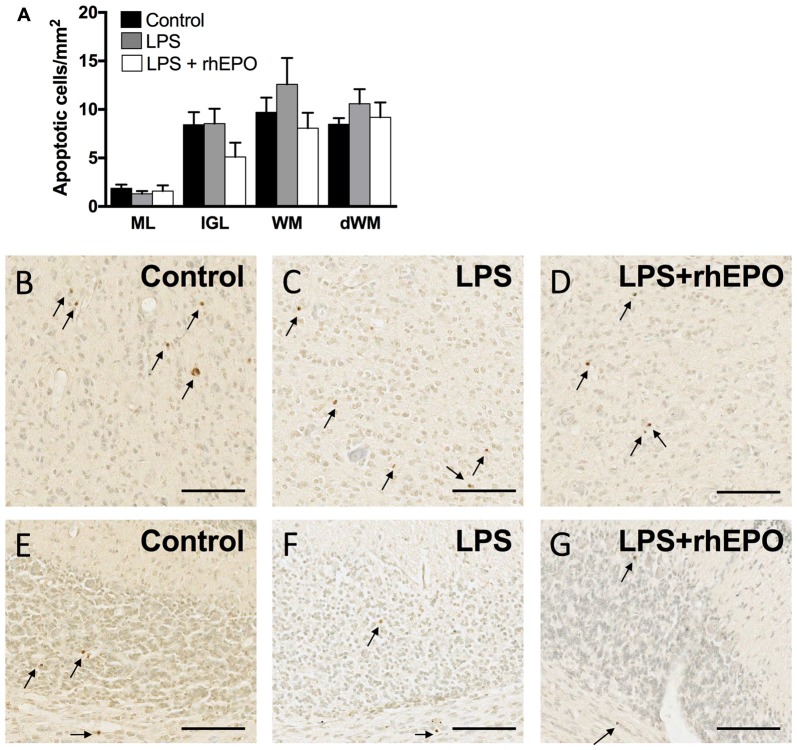
Apoptosis. **(A)** The areal density of TUNEL-positive apoptotic cells in the ML, IGL, lobular WM and deep WM in saline-treated Control (black bars), LPS-treated (gray bars) and LPS + rhEPO-treated (white bars) fetal sheep. TUNEL positive cells (brown cells indicated by arrows) in the deep WM of **(B)** saline-treated (Control), **(C)** LPS-treated and **(D)** LPS + rhEPO-treated fetal sheep and in lobule VIII of **(E)** saline-treated (Control), **(F)** LPS-treated and **(G)** LPS + rhEPO-treated fetal sheep. Scale bar = 100 μm.

There were no differences in the density of TUNEL-positive apoptotic cells between control, LPS and LPS + rhEPO animals in the ML, IGL, lobular WM or deep WM (Figure 6A). Representative images of TUNEL-positive cells are presented in Figures 6B–G.

## Discussion

This is the first study to report that rhEPO can reduce LPS-induced inflammatory effects in the developing fetal cerebellum. Our major findings were that rhEPO treatment reduced: (1) microgliosis in the lobular WM; (2) the increase in thickness of the proliferative and post-mitotic zones of the EGL; and (3) the increase in granule cell density in the IGL, induced by LPS. These results are in accordance with our findings of the neuroprotective effects of rhEPO on LPS-induced injury in the cerebral hemispheres and major axonal tracts (Rees et al., 2010). In both studies, neuroprotection occurred without any significant exacerbation or amelioration of the physiological effects of LPS on the fetus (Rees et al., 2010). Our findings are consistent with observations that rhEPO has been shown to be neuroprotective in the cerebellum against hypoxia-ischemia (Iwai et al., 2007) and umbilical cord occlusion-induced fetal hypoxemia (Traudt et al., 2013).

In accordance with the only study to examine the effects of chorioamnionitis in the human infant cerebellum (Limperopoulos et al., 2005), we did not find any gross injury such as focal infarcts or hemeorrhage in the LPS-exposed cerebellar cortex or WM in this, or our previous study (Duncan et al., 2002). Strackx et al. (2012) also reported no overt cerebellar damage in a similar sheep model of inflammation although Dean et al. (2009) found some focal lesions in animals exposed to LPS at an earlier gestational age (93-96 DGA). Studies that have characterized LPS-induced WM injury at the cellular level report finding microgliosis, astrogliosis, increased apoptosis, decreased maturation of oligodendrocytes, and compromised blood-brain barrier integrity (Hutton et al., 2007; Dean et al., 2009; Gavilanes et al., 2009; Strackx et al., 2012). In the present study, we found that LPS-induced microgliosis but not astrogliosis in the cerebellar WM, and that there was no evidence of an increase in apoptosis in any cerebellar region. We acknowledge that astrogliosis might have been evident at earlier time-points, as we know that it precedes microgliosis in this ovine model (Duncan et al., 2002). Blood-brain barrier integrity or markers of oligodendrocyte development were not investigated in the present study. Any inconsistencies between the degree of cerebellar injury found in our study and those reported above are most likely due to the route of delivery of the LPS (intra-amniotic vs. intravenous), different doses of LPS, the developmental stage at which LPS was administered, or the duration of exposure to inflammation.

The significant increase in the density of granule cells in the IGL of LPS-exposed animals was unexpected but is in accordance with the findings of Strackx et al. (2012) who reported an increase in total granule cell number in the IGL 30 days after LPS exposure. Although we were not able to calculate total neuronal numbers, the finding that the cross-sectional area of the IGL was not different from controls suggests that the increase in density might have resulted in an increase in total granule cell numbers. Granule cell numbers could increase as a result of increased proliferation in the EGL, increased rate of migration from the EGL to the IGL, decreased apoptosis, increased cell survival, or a decrease in the growth of granule cell processes resulting in a higher packing density. Here we show a significant increase in the thickness of the proliferative and post-mitotic zones of the EGL in sheep exposed to LPS compared to controls. This is possibly due to the increase in granule cell proliferation and the concomitant increase in cells entering the post-mitotic phase of the cycle. There appears to be no change in the rate at which granule cells are leaving the EGL to migrate through the ML, as suggested by the lack of observed differences in the density of granule cells in the ML. Further analysis, such as live imaging of granule cell migration in cerebellar slices in the presence of LPS would be required to confirm this. We did not observe any qualitative differences between groups in the level of apoptosis in the EGL. Thus our results suggest that LPS increases proliferation of granule cells in the EGL, resulting in an increase in granule cell density in the IGL. The mechanisms underlying this observation are likely to be multifactorial and require investigation. Any alterations to the normal program of cellular development in the cerebellum could result in altered connectivity and subsequent adverse functional outcomes.

Although it seems counter-intuitive that inflammation results in an increase in neuronal density, given the well-characterized role of pro-inflammatory cytokines in promoting neuronal cell death (Dean et al., 2009; Gavilanes et al., 2009), a similar result was observed in mice after maternal infection, with increased pyramidal and granule cells in the hippocampus (Golan et al., 2005). The role of cytokines in the central nervous system is complex as there is evidence that the cytokine TNF-alpha, which increases 3.5-fold after LPS exposure in the ovine model used in the current study (Rees et al., 2010), can enhance neuronal growth and survival during development depending on which TNF-α receptor is activated (Yang et al., 2002). Similarly IL-6, which is also increased 4-fold after LPS exposure in this ovine model (Duncan et al., 2002), has a positive influence on cell survival, at least *in vitro* (Hama et al., 1989).

Although granule cell numbers were clearly altered by LPS exposure, Purkinje cell numbers were not affected; a similar result has been reported previously by our group (Duncan et al., 2002) and by Strackx et al. (2012). The different effects of LPS on these cell populations could be due to their developmental stage at the time of the LPS exposure; granule cells are still in the phase of proliferation and migration while Purkinje cells have reached their mature numbers and distribution before 120 DGA in sheep (Rees and Harding, 1988).

### rhEPO Treatment Reduces Microgliosis in the White Matter

The reduction of microgliosis in the lobular WM following 9 days of treatment suggests that rhEPO is able to ameliorate the inflammatory action of LPS, as we have shown previously for the WM in the cerebral hemispheres, brain stem and optic nerves (Rees et al., 2010). It is not possible to determine whether the LPS-induced microgliosis resulted from proliferation of resident microglia or invasion by peripheral macrophages in response to inflammatory molecules in the brain in the present study. We note that LPS-induced damage is not as extensive in the cerebellum as it is in the other regions of the CNS however, rhEPO clearly reduced the microglial response either directly or indirectly by protecting WM from damage.

### rhEPO Reduces the LPS-Induced Increase in Granule Cell Density

The significant increase in the width of the proliferative and post-mitotic zones of the EGL (both ~25%) and in granule cell density in the IGL (~35%) induced by LPS exposure was reduced by rhEPO treatment. This was an unexpected effect of rhEPO given the well characterized role of EPO in promoting neurogenesis and neuronal survival, in addition to promoting proliferation of other cell types (reviewed in Chen et al., 2006). We know that rhEPO does not reduce the levels of the pro-inflammatory cytokine TNF-alpha in LPS-exposed fetal sheep (Rees et al., 2010) so any involvement by cytokines is not clear-cut. rhEPO signaling might act, at least in part, via the Purkinje cells in the developing sheep cerebellum (Castillo-Meléndez et al., 2005), although any mechanism of rhEPO’s action on specific cerebellar cell types remains unknown. Clearly further research is warranted into the mechanisms involved. Whatever the effects of inflammation on the normal developmental processes in the cerebellum, here we show that rhEPO ameliorates these changes, returning granule cell density to control levels.

A limitation of our study, as well as previous studies on inflammation in the developing cerebellum (Hutton et al., 2007; Dean et al., 2009; Strackx et al., 2012), is that we only examined one time point following LPS administration. Although beyond the scope of the present study, it would be instructive to examine the effects of rhEPO and LPS on the developing cerebellum in the short-, medium- and long-term.

## Conclusion

Although few studies have examined the effects of inflammation on the developing cerebellum in human infants, there is evidence from animal studies demonstrating injury and altered development following exposure to LPS. In the present study, we have demonstrated for the first time that rhEPO can ameliorate effects of inflammation of the developing cerebellum. The inflammatory response resulted in microgliosis in the cerebellar WM, an increase in the proliferative and post-mitotic zones in the EGL and an increase in the density of granule cells in the IGL, all of which were restored to control levels by rhEPO. Our data suggest that rhEPO is neuroprotective against inflammation-induced microgliosis and altered development in the cerebellum. In conjuction with our previous findings of the neuroprotective potential of rhEPO in reducing fetal cerebral, brain stem and optic nerve damage after LPS exposure (Rees et al., 2010), they support the possibility of improved neurodevelopmental outcomes with the use of this treatment for the compromised immature brain (Neubauer et al., 2010; O’Gorman et al., 2015).

## Author Contributions

ARAM, SR, RH, RDM, SBH and MT: conception and design of study; ARAM, NH, SR, RH, RDM and MT: acquisition and analysis of data; ARAM, NH, SR, RH, SBH and MT: interpretation of data; ARAM, NH, SR, RH, RDM, SBH and MT: drafting the work or revising it critically for important intellectual content; AM, NH, SR, RH, RDM, SBH and MT: final approval of the version to be published.

## Conflict of Interest Statement

The authors declare that the research was conducted in the absence of any commercial or financial relationships that could be construed as a potential conflict of interest. The handling Editor declared a shared affiliation, though no other collaboration, with several of the authors (ARAM, RH, RDM, SBH) and states that the process nevertheless met the standards of a fair and objective review.

## Footnotes

^1^http://fiji.sc/Fiji
